# Supplementary material for: Pushing the resolution limit by correcting the Ewald sphere effect in single-particle Cryo-EM reconstructions
Source: Nat Commun. 2018 Apr 19;9:1552. doi: 10.1038/s41467-018-04051-9 (PMC5908801; doi:10.1038/s41467-018-04051-9)
Supplement: Supplementary file 1 — Supplementary Information [file 41467_2018_4051_MOESM1_ESM.pdf]

Pushing the resolution limit by correcting the Ewald sphere effect in single-particle Cryo-EM reconstructions

Zhu et al.

Supplementary Information

Supplementary Figure 1

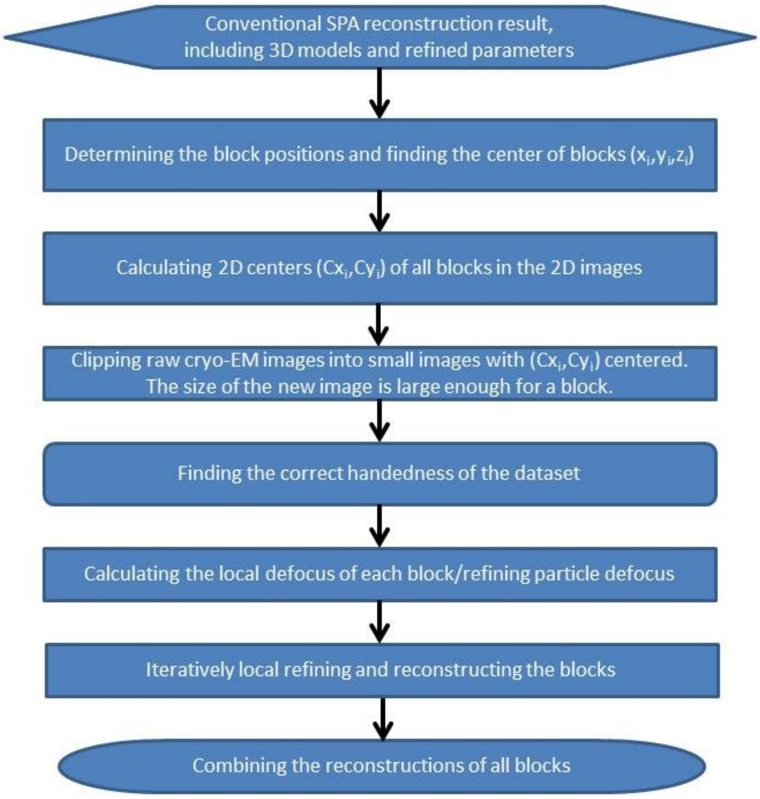

Supplementary Figure 1. The overall work flow of the method.

The area of each block and the center of the block are defined on the 3D reconstruction map achieved by a conventional SPA reconstruction method. The center of the block on the 2D images can be calculated using the translational and rotational parameters of the 2D images. The densities of the block in the 2D image are centered and clipped to create the sub-image. The particle defocus of each virus can be obtained by fitting the Thon ring signal or by a refinement procedure. The defocus variation between the center of the virus and the center of a block along the Z axis can be calculated. The local defocus of a block is the sum of the defocus variation and the particle defocus. The structure of a block can be reconstructed by sub-images with the rotational parameters and the local defocus. In the sub-image, the densities of a block are normally overlapped with the densities from other blocks. We found the structure of the block can be effectively refined using a local search of the rotational and translational parameters without density subtraction. However, it is a bad idea to do global refinement, the resolution of the final map would be significantly worse probably due to the overlapped densities. After all the blocks being refined, the densities of these blocks are combined to form a virus.

Supplementary Figure 2

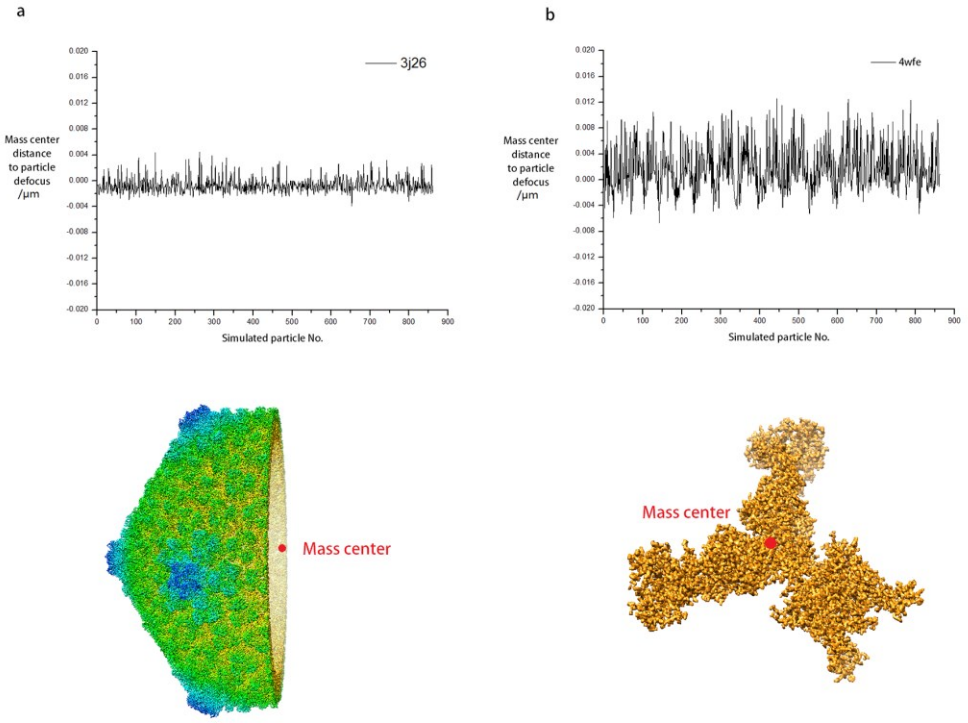

Supplementary Figure 2. The distance from the plane of measured defocus on simulated images to the plane of centre of mass along the Z axis.

a, In a protein complex with a spherical shape such as icosahedral viruses, the centre of mass of the virus is located at the center of the virus. The differences between the measured defoci in simulated images with different projected Euler angles and the particle defocus are shown. b, The differences between the measured defoci in simulated images with different projected Euler angles and the particle defocus of a non-spherical object are shown.

Supplementary Figure 3

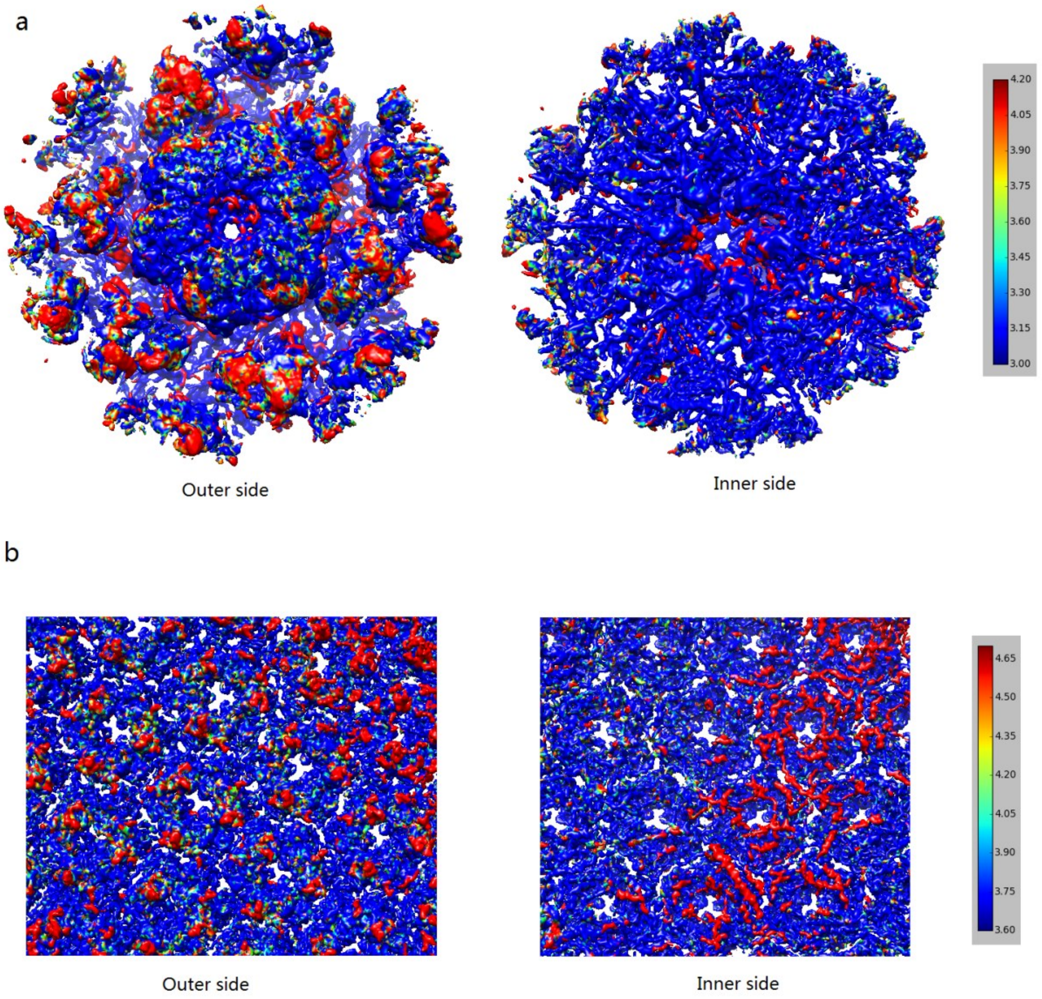

Supplementary Figure 3. Local resolution calculated by ResMap[1].

a, The map contains one hexon and six triplex of HSV-2 capsid. b, The map contains part of an asymmetric unit of PBCV-1 capsid. The minimum resolution calculated by ResMap is about 2.2 times of pixel size. As a result, the minimum resolution available in b is 3.6 Å rather than 3.5 Å.

Supplementary Table 1
Cryo-EM data collection, refinement and validation statistics

	HSV-2	PBCV-1
**Data collection and processing**		
Voltage (kV)	300	300
Defocus range (μm)	1.0-4.0	1.0-3.0
Pixel size (Å)	1.38	1.62
Symmetry imposed	EMAN icos & C1	EMAN icos & C1
Initial particle images (no.)	53620	12974
Final particle images (no.)	34695	12974
Map resolution (Å)<br>FSC threshold	Sym=I, 4.0. Sym=C1, 3.1,<br>with FSC-0.143	Sym=I, 4.2. Sym=C1, 3.5,<br>with FSC-0.143
Map resolution range (Å)	3.0-4.2	3.5-4.7

Supplementary Note 1

Our block-based reconstruction method contains preprocessing of the extracted images by home-made Python scripts. Local refinement and 3D reconstruction can be performed by using EMAN[2], JSPR[3], RELION[4] or most other current available cryo-EM software packages after implementation. The reconstructions of blocks can be combined by EMAN-library based home-made programs. All needed home-made scripts and programs are available at

https://github.com/homurachan/Block-based-recontruction

Supplementary Reference

1. Kucukelbir, A., Sigworth, F.J. & Tagare, H.D. Quantifying the local resolution of cryo-EM density maps. *Nat Methods* **11**, 63-5 (2014).
2. Tang, G. et al. EMAN2: an extensible image processing suite for electron microscopy. *J Struct Biol* **157**, 38-46 (2007).
3. Guo, F. & Jiang, W. Single particle cryo-electron microscopy and 3-D reconstruction of viruses. *Methods Mol Biol* **1117**, 401-43 (2014).
4. Scheres, S.H. RELION: implementation of a Bayesian approach to cryo-EM structure determination. *J Struct Biol* **180**, 519-30 (2012).
